# Conjugative Botulinum Neurotoxin-Encoding Plasmids in *Clostridium botulinum*

**DOI:** 10.1371/journal.pone.0011087

**Published:** 2010-06-11

**Authors:** Kristin M. Marshall, Marite Bradshaw, Eric A. Johnson

**Affiliations:** Department of Bacteriology, College of Agriculture and Life Sciences, University of Wisconsin, Madison, Wisconsin, United States of America; Max Planck Institute for Infection Biology, Germany

## Abstract

**Background:**

*Clostridium botulinum* produces seven distinct serotypes of botulinum neurotoxins (BoNTs). The genes encoding different subtype neurotoxins of serotypes A, B, F and several dual neurotoxin-producing strains have been shown to reside on plasmids, suggesting that intra- and interspecies transfer of BoNT-encoding plasmids may occur. The objective of the present study was to determine whether these *C. botulinum* BoNT-encoding plasmids are conjugative.

**Methodology/Principal Findings:**

*C. botulinum* BoNT-encoding plasmids pBotCDC-A3 (strain CDC-A3), pCLJ (strain 657Ba) and pCLL (strain Eklund 17B) were tagged with the erythromycin resistance marker (Erm) using the ClosTron mutagenesis system by inserting a group II intron into the neurotoxin genes carried on these plasmids. Transfer of the tagged plasmids from the donor strains CDC-A3, 657Ba and Eklund 17B to tetracycline-resistant recipient *C. botulinum* strains was evaluated in mating experiments. Erythromycin and tetracycline resistant transconjugants were isolated from donor∶recipient mating pairs tested. Transfer of the plasmids to the transconjugants was confirmed by pulsed-field gel electrophoresis (PFGE) and Southern hybridizations. Transfer required cell-to-cell contact and was DNase resistant. This indicates that transfer of these plasmids occurs via a conjugation mechanism.

**Conclusions/Significance:**

This is the first evidence supporting conjugal transfer of native botulinum neurotoxin-encoding plasmids in *C. botulinum*, and provides a probable mechanism for the lateral distribution of BoNT-encoding plasmids to other *C. botulinum* strains. The potential transfer of *C. botulinum* BoNT-encoding plasmids to other bacterial hosts in the environment or within the human intestine is of great concern for human pathogenicity and necessitates further characterization of these plasmids.

## Introduction

*Clostridium botulinum* is classified as an anaerobic, gram-positive, sporeforming organism that produces an extraordinarily lethal toxin designated botulinum neurotoxin (BoNT). BoNTs are categorized into seven different serotypes listed A–G, and multiple subtype BoNTs have been discovered and continue to be identified, especially among serotypes A, B, E and F [1], [2], [3], [4], [5], [6].

This highly heterogeneous species of *C. botulinum* is divided into four groups based on metabolic, physiological and genetic properties [7]. Historically, these neurotoxigenic clostridia have been classified as *C. botulinum* based on their capacity to produce BoNTs, but many researchers believe that the four clades represent separate species of clostridia [2], [8], [9]. Group I contains proteolytic strains of serotypes A, B and F, whereas Group II encompasses nonproteolytic strains of serotypes B, E and F. Unlike proteolytic strains, nonproteolytic strains lack the ability to digest meat and milk proteins and rely on exogenous proteases for the proteolytic nicking of the neurotoxin into its active di-chain form [10]. *C. botulinum* serotype C and D strains are clustered in Group III and strains of *C. botulinum* serotype G, also referred to as *Clostridium argentinense*, are included in Group IV.

BoNT is the etiologic agent of botulism, a paralytic disease resulting from the inhibition of neurotransmitter release at the neuromuscular junction [11]. There are several forms of botulism, and infant and foodborne represent the majority of botulism cases reported in the U. S. [12]. Despite the unsavory reputation of BoNTs as a deadly poison and as a potential bioterrorism agent, their use in treatment for numerous hyperactive muscle disorders has been widely demonstrated [13].

Although the genes encoding BoNTs of serotypes C, D and G have been established to be associated with extrachromosomal elements [14], [15], the location of genes encoding serotypes A, B, E and F were believed to be located on the chromosome from earlier analyses of total genomic DNA [16]. Recently, strains of serotype A, proteolytic and nonproteolytic strains of serotype B, and dual neurotoxin-producing Ba, Ab and Bf strains have been shown to harbor neurotoxin genes on very large plasmids [6], [17], [18]. Interestingly, in dual neurotoxin-producing strains of Ba, Ab and Bf subtypes analyzed thus far, it appears that both neurotoxin genes are usually located on the same plasmid [6], [18]. Plasmids identified in proteolytic strains of *C. botulinum* range in size from ∼150 to 270 kb and several plasmids found in serotypes A and B and dual neurotoxin producing Ba and Bf strains have been shown to be highly conserved, yet they carry different neurotoxin subtype genes [6], [8]. BoNT-encoding plasmids seem to be more prevalent among strains of serotype B than other serotypes [6], [18], [19]. Unlike the large plasmids observed in proteolytic serotype B strains, plasmids found in nonproteolytic B strains are consistently smaller (∼48 kb) and share no homology with plasmids of proteolytic *C. botulinum* strains [6], [8].

These observations led to the hypothesis that BoNT-encoding plasmids are the likely vehicles for the dissemination of neurotoxin genes and are possibly transmissible at least between strains of *C. botulinum* and perhaps to strains of closely related clostridial species such as *C. baratii* and *C. butyricum*. Isolates of *C. baratii* and *C. butyricum* have been found to produce BoNT/F and BoNT/E respectively [12], but how these strains acquired the neurotoxin genes is presently unknown.

To test the hypothesis that *C. botulinum* plasmids are transmissible, the BoNT-encoding plasmids pBotCDC-A3 (proteolytic subtype A3 strain CDC-A3), pCLJ (proteolytic subtype A4/bivalent B strain 657Ba) and pCLL (nonproteolytic serotype B strain Eklund 17B) were tagged using the ClosTron mutagenesis system [20], [21]. The BoNT/A3 subtype gene, *bont/A3* (pBotCDC-A3), the bivalent BoNT/B gene, *bont/bvB* (pCLJ), and the nonproteolytic BoNT/B gene *bont/npB* (pCLL) were insertionally inactivated by insertion of the group II intron containing the erythromycin resistance marker. The tagged plasmids were used in mating experiments with the recipient *C. botulinum* strain LNT01. *C. botulinum* strain LNT01 is a transposon Tn*916* mutant of the parent *C. botulinum* subtype A1 strain 62A [22], [23]. Upon integration of the transposon the BoNT/A1 neurotoxin gene cluster and regions flanking the cluster were deleted resulting in a nontoxigenic *C. botulinum* strain that carries the tetracycline resistance determinant. In this study we report the first demonstration of native BoNT-encoding plasmid transfer from an A3 subtype strain, an A4/bivalent B subtype strain and a nonproteolytic serotype B strain to a nontoxigenic mutant of a subtype A1 strain.

## Results

Plasmids carrying neurotoxin genes have been identified in some proteolytic and nonproteolytic *C. botulinum* strains of serotypes A and B, and in bivalent subtypes Ba, Bf and Ab [6], [8], [17], [18], [24]. We hypothesize that BoNT-encoding plasmids are capable of intra- and interspecies transfer, since they are present in a variety of proteolytic and nonproteolytic *C. botulinum* strains. To test our hypothesis that *C. botulinum* BoNT-encoding plasmids are transmissable, mating experiments were conducted between donors harboring a BoNT-encoding plasmid and a nontoxigenic *C. botulinum* strain LNT01 as the recipient. *C. botulinum* strains CDC-A3 and 657Ba, which harbor large plasmids, pBotCDC-A3 (267 kb) and pCLJ (270 kb), respectively, were selected as donors to represent proteolytic strains. As a representative of nonproteolytic *C. botulinum* serotype B, strain Eklund 17B containing plasmid pCLL (48 kb) was chosen as a donor strain. The recipient strain LNT01 was selected because it is nontoxigenic, and it contains a tetracycline resistance marker due to presence of the transposon Tn*916* on the genome [22], [23]. To ascertain transfer of BoNT-encoding plasmids from the donor to the recipient strain, the plasmids were tagged with an erythromycin resistance gene using the ClosTron mutagenesis system. Thus, positive selection of transconjugants was facilitated by the presence of the tetracycline resistance determinant in combination with erythromycin resistance provided by the tagged plasmids.

### Plasmid tagging using ClosTron

**Figure 1:**
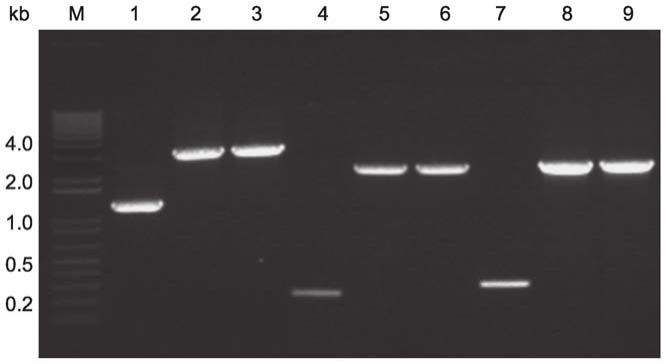
Confirmation of tagging *C. botulinum* BoNT-encoded plasmids pBotCDC-A3-Erm (strain CDC-A3), pCLJ-Erm (strain 657Ba) and pCLL-Erm (strain Eklund 17B) by PCR analysis. PCR products of wild-type *C. botulinum* strains CDC-A3 (Lane 1), 657Ba (Lane 4) and Eklund 17B (Lane 7) and two erythromycin resistant, thiamphenicol sensitive clones of each of CDC-A3 (Lanes 2 and 3), 657Ba (Lanes 5 and 6) and Eklund 17B (Lanes 8 and 9) strains; 1 kb Plus ladder (Invitrogen) (Lane M).

The neurotoxin genes, *bont/A3* of plasmid pBotCDC-A3 (strain CDC-A3), *bont/bvB* of plasmid pCLJ (657Ba), and *bont/npB* of plasmid pCLL (strain Eklund 17B) were insertionally inactivated using the ClosTron mutagenesis system [20], [21]. The potential intron target sites within each neurotoxin gene were identified using the computer algorithm at the group II intron (TargeTron) design site provided by Sigma-Aldrich (St. Louis, MO). The target sites chosen for *bont/A3*, *bont/bvB* and *bont/npB* were between nucleotides 580 and 581, 381 and 382, and 420 and 421 on the sense strands, respectively. Each re-targeted intron was amplified by PCR and cloned into the ClosTron vector pMTL007C-E2 between restriction sites *Hind*III and *BsrG*I [21] resulting in constructs, pMTL007C-E2:Cbo:*bont/A*-580s, pMTL007C-E2:Cbo:*bont/bvB*-381s, and pMTL007C-E2:Cbo:*bont/npB*-420s. The constructs were transferred to their respective wild-type strains CDC-A3, 657Ba and Eklund 17B by conjugation from the *E. coli* donor strain CA434. Following matings, the cells were plated onto agar containing thiamphenicol to select for *C. botulinum* clones harboring the ClosTron vector. Thiamphenicol resistant transconjugants of *C. botulinum* containing the ClosTron vector were then plated onto agar supplemented with erythromycin for selection of intron integrants, since the erythromycin resistance gene is restored upon integration of the group II intron [20], [21]. Next, erythromycin resistant clones were screened for the loss of the intron vector by replica plating, then erythromycin resistant and thiamphenicol sensitive clones were selected and further analyzed by PCR to determine whether the intron had integrated into its desired target site. The gene specific PCR primers (Table 1) were designed to anneal to regions flanking the insertion site for each neurotoxin gene in order to amplify the entire insertion element. Insertion of the re-targeted introns into either the *bont/A3*, *bont/bvB* or *bont/npB* genes was confirmed by PCR analysis (Fig. 1). PCR amplification of the DNA from the wild type CDC-A3 strain using the *bont/A3* gene specific primers A3KMCT1 and A3KMCT2 produced a PCR product of 1,264 bp (Fig. 1, Lane 1), whereas a DNA fragment of 3,044 bp was observed in the CDC-A3 transconjugant clones analyzed (Fig. 1, Lanes 2 and 3), indicating integration of the intron element (∼1.8 kb) into the target gene. Similarly, amplification of the 657Ba and Eklund 17B transconjugant clones (Fig. 1 Lanes 5 and 6, and Lanes 8 and 9, respectively) using *bont/bvB* and *bont/npB* gene specific primers yielded expected PCR products that exhibited an ∼1.8 kb increase in size compared to the PCR fragments generated from the wild type 657Ba (Fig. 1, Lane 4) and Eklund 17B (Fig. 1, Lane 7). These results confirmed that the re-targeted introns containing the erythromycin resistance determinant *ermB* were inserted into the *bont/bvB* and *bont/npb* (Fig. 1). Furthermore, the PCR fragments amplified from the tagged BoNT-encoding plasmids were sequenced and it was confirmed that the introns had inserted correctly into the chosen target sites within the neurotoxin genes in all three plasmids.

**Table 1:** Oligonucleotide primers used in this study.

Oligonucleotide Primer	Sequence (5′ - 3′)
580|581s-IBS	AAAAAAGCTTATAATTATCCTTACAGATCTTACATGTGCGCCCAGATAGGGTG
580|581s-EBS1d	CAGATTGTACAAATGTGGTGATAACAGATAAGTCTTACATTTTAACTTACCTTTCTTTGT
580|581s-EBS2	TGAACGCAAGTTTCTAATTTCGGTTATCTGTCGATAGAGGAAAGTGTCT
381|382s-IBS	AAAAAAGCTTATAATTATCCTTAGTTCCCCTCGAAGTGCGCCCAGATAGGGTG
381|382s-EBS1d	CAGATTGTACAAATGTGGTGATAACAGATAAGTCCTCGAAGATAACTTACCTTTCTTTGT
381|382s-EBS2	TGAACGCAAGTTTCTAATTTCGATTGGAACTCGATAGAGGAAAGTGTCT
420|421S-IBS	AAAAAAGCTTATAATTATCCTTAACTGTCAATAAAGTGCGCCCAGATAGGGTG
420|421S-EBS1d	CAGATTGTACAAATGTGGTGATAACAGATAAGTCAATAAATTTAACTTACCTTTCTTTGT
420|421S-EBS2	TGAACGCAAGTTTCTAATTTCGATTACAGTTCGATAGAGGAAAGTGTCT
EBS Universal	CGAAATTAGAAACTTGCGTTCAGTAAAC
A3KMCT1	GAGATCCTGTAAATGGTGTTGATATTGC
A3KMCT2	GGTATTATCCCTCTTACACATAGCAGC
BVBFCT4	CAAACAATGATCAAGTTATTTAATAG
BVBRCT4	TCATTTAAAACTGGCCCAGG
NPBFCT11	CAAATCAAAACCATTGGGTGAAAAG
NPBRCT11	CTGGACAAAATTTCATTTGCATTATACCCC
AnyBF	CAGGAGAAGTGGAGCGAAAAAAAG
AnyBR	TGGTAAGGAATCACTAAAATAAGAAGC
Erm-F	CCGATACCGTTTACGAAATTGGAACAGG
Erm-R	TTATTTCCTCCCGTTAAATAATAGATAACT
pMTL007-R1	AGGGTATCCCCAGTTAGTGTTAAGTCTTGG

**Figure 2:**
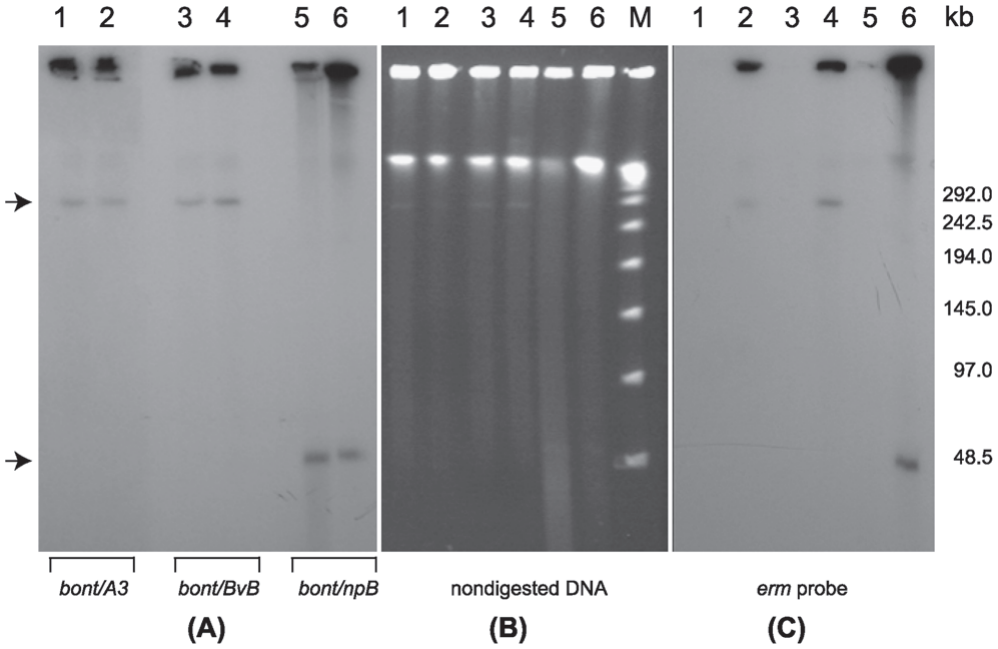
Confirmation of tagging *C. botulinum* BoNT-encoding plasmids pBotCDC-A3-Erm (strain CDC-A3), pCLJ-Erm (strain 657Ba) and pCLL-Erm (strain Eklund 17B) by PFGE and Southern hybridization analysis. (**B**) Ethidium bromide stained PFGE of nondigested DNA samples from *C. botulinum* strains: wild type CDC-A3 (Lane 1), CDC-A3580s1 (Lane 2), wild type 657Ba (Lane 3), 657Ba-CT4 (Lane 4), wild type Eklund 17B (Lane 5) and Eklund17B-CT11 (Lane 6); Lambda PFG Marker (Lane M), (New England Biolabs). The position of BoNT-encoding plasmids is indicated with arrows. (**A**) Southern hybridization with the *bont/A3* probe (Lanes 1 and 2); the *bont/bvB* probe (Lanes 3 and 4) and the *bont/npB* probe (Lanes 5 and 6); (**C**) Southern hybridization with the *ermB* probe. PFGE conditions: 6V/cm, 12°C, 1–20 s pulse time, 24 h.

To verify that the plasmids were tagged, pulsed-field gel electrophoresis (PFGE) of nondigested DNA samples from the wild type strains CDC-A3, 657Ba and Eklund 17B, and the clones carrying the tagged plasmids pBotCDC-A3-Erm, pCLJ-Erm and pCLL-Erm, was performed followed by Southern hybridization analyses using probes specific to *ermB* and the respective neurotoxin genes. Hybridization signals were observed with the plasmid bands in all strains using the neurotoxin gene probes (Fig. 2). Hybridization of the *ermB* probe was detected with the tagged plasmid clones but not with the wild type strains, indicating that the plasmids were successfully tagged with the ErmB-RAM. The resultant strains with their tagged plasmids CDC-A3580s1 (pBotCDC-A3-Erm), 657BaCT4 (pCLJ-Erm) and Eklund 17BCT11 (pCLL-Erm) were used as the donors in the mating experiments.

### Mating experiments

Separate mixed plate matings between each donor strain, CDC-A3580s1 (pBotCDC-A3-Erm), 657BaCT4 (pCLJ-Erm), and Eklund 17BCT11 (pCLL-Erm) and recipient strains LNT01 and Hall A-*hyper*/Tn*916* mutant were performed inside an anaerobic chamber on solid 4% agar TYG media for 12 h. Initially, strain LNT01 was used as the recipient to determine if plasmids pBotCDC-A3-Erm, pCLJ-Erm, and pCLL-Erm could be transferred to a recipient *C. botulinum* strain. Several mating experiments were performed to optimize the mating conditions to establish the transfer frequencies. Since similar transfer frequencies were observed when matings were performed for 12 or 24 h (data not shown); all subsequent bacterial mating experiments were incubated for 12 h. The mating pairs between proteolytic strains were performed at their optimal growth temperature of 37°C. Matings of the nonproteolytic serotype B donor strain Eklund 17BCT11 and the recipient strain LNT01 were performed at 30°C, which is the optimal growth temperature for nonproteolytic *C. botulinum* strains, since higher transfer frequencies were observed at this temperature (data not shown). Three different donor to recipient ratios (5∶1, 1∶1 and 1∶5) were tested, the donor∶recipient (D∶R) ratio of 1∶1 yielded the highest transfer frequencies. After the mating conditions were established in LNT01 the same experimental parameters were used to evaluate the transfer frequencies of *C. botulinum* plasmids into *C. botulinum* strain Hall A-*hyper*/Tn*916* mutant.

Transconjugants were selected by plating the mating mixtures onto TYG agar supplemented with erythromycin (selection of Erm-plasmid) and tetracycline (selection of recipient strain LNT01 or Hall A-*hyper*/Tn*916*. To determine the number of donors and recipients the mating mixtures were also plated onto TYG containing either erythromycin (donors) or tetracycline (recipients). The number of donor cells and recipients varied with respect to the mating pairs (Table 2). The transfer frequency was calculated as the number of transconjugants per recipient or donor depending on which strain had the highest CFU/ml.

**Table 2:** Transfer of *C. botulinum* BoNT-encoding plasmids to recipient strains LNT01 and Hall A-*hyper*/Tn*916*.

		Transfer Frequency	
		Recipient	
Donor	Plasmid	LNT01	Hall A-*hyper*/Tn*916*
CDC-A3580	pBotCDC-A3-Erm	1.5×10^−8^±1.2×10^−8*a*^	1.8×10^−6^±9.4×10^−7*b*^
657Ba-CT4	pCLJ-Erm	1.4×10^−6^±1.1×10^−6*a*^	1.7×10^−5^±1.2×10^−5*a*^
Eklund 17BCT11	pCLL-Erm	1.5×10^−7^±1.4×10^−7*a*^	4.5×10^−7^±2.8×10^−7*a*^

Transfer frequencies were calculated as the number of tranconjugants per *^a^*recipient or *^b^*donor and are reported as the averages of at least three replicate experiments.

The transfer frequency values are displayed in Table 2. Overall, the plasmid transfer frequencies were lower than those reported for plasmids found in strains of *Clostridium perfringens* [25], [26], [27]. The conjugation frequencies for pBotCDC-A3-Erm and of pCLJ-Erm increased markedly when Hall A-*hyper*/Tn*916* was used as the recipient. Similar conjugation frequencies of plasmid pCLL from the nonproteolytic strain Eklund 17B were observed when either strain LNT01 or Hall A-hyper/Tn*916* was used as recipient (Table 2).

Pre-incubation of the donor cells with DNaseI, by combined addition of DNaseI to the agar medium and to the mating mixtures did not inhibit plasmid transfer, and the transfer frequencies were similar to that of matings in which DNaseI was not added. Furthermore, no transductants were obtained in matings performed with the filtered culture supernatants of each donor strain and the whole cell culture of the recipient strain LNT01. Importantly, no transconjugants were obtained when matings were performed in which the donors and recipients were separated by a 0.45 µm nitrocellulose membrane.

### Confirmation of BoNT-encoding plasmid transfer

**Figure 3:**
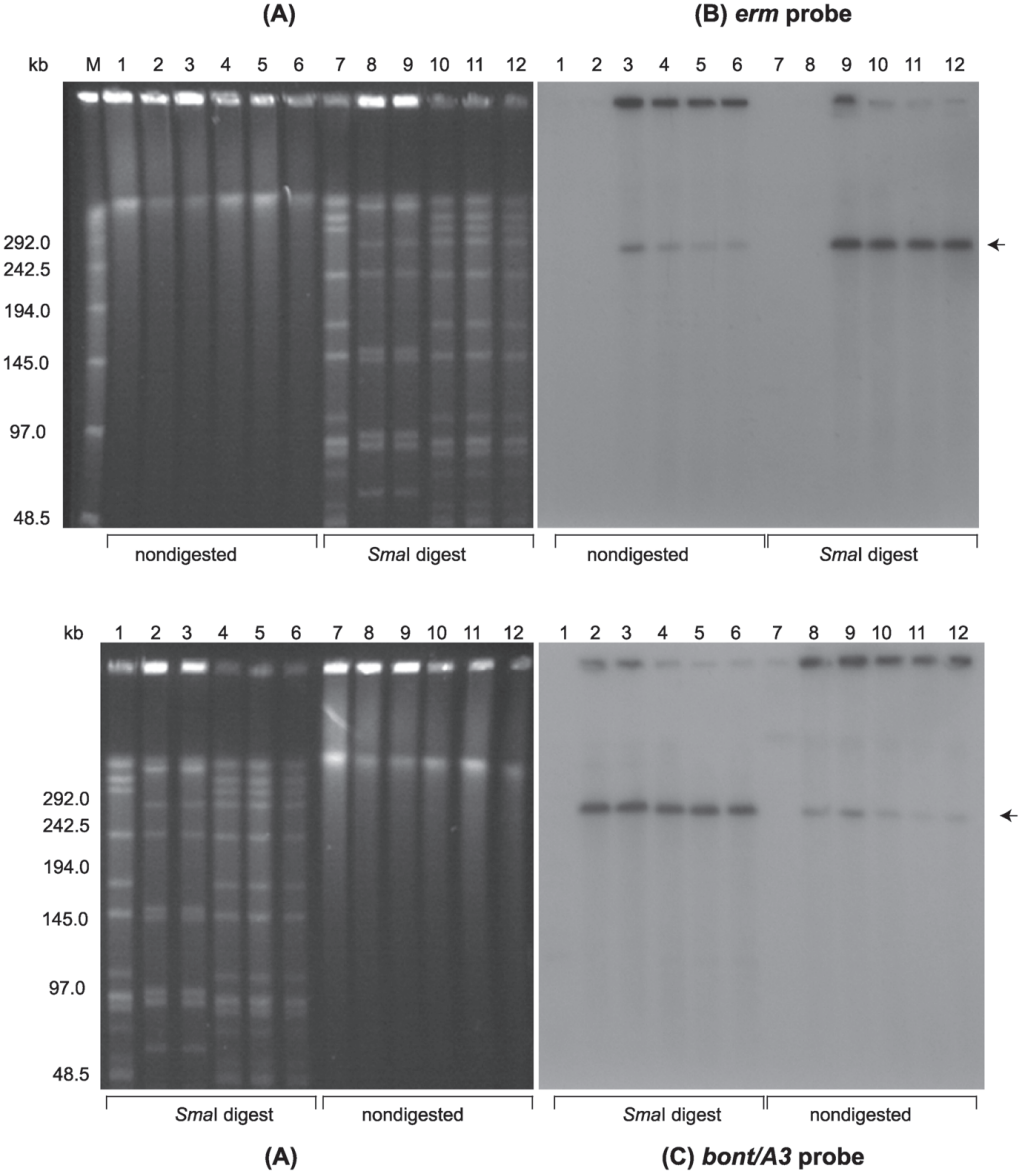
Confirmation of plasmid pBotCDC-A3-Erm transfer from *C. botulinum* strain CDC-A3580s1 to strain LNT01 by PFGE and Southern hybridization analysis. (**A**) Ethidium bromide stained PFGE of *C. botulinum* DNA samples: *Sma*I digested DNA of *C. botulinum* strain LNT01 (Lanes 1 and 7), CDC-A3 wild type (Lanes 2 and 8), CDC-A3580s1 (Lanes 3 and 9), and LNT01 transconjugants (pBotCDC-A3-Erm) (Lanes 4–6 and 10–12); Lanes 1–6, nondigested DNA samples; Lanes 7–12, *Sma*I digested DNA samples. Lambda PFG Marker (Lane M), New England Biolabs. The position of the pBotCDC-A3 plasmid is indicated with an arrow. Southern hybridization with: (**B**) the *ermB* probe, and (**C**) the *bont/A3* probe. PFGE conditions: 6V/cm, 12°C, 1–26 s pulse time, 24 h.

PFGE was performed using nondigested samples and samples digested with restriction enzymes designed to linearize each BoNT-encoding plasmid. PFGE analysis of digested samples allowed us to use the unique restriction banding patterns of the strains as a genetic screen to visually determine whether the plasmids were transferred to the recipient strains. PFGE analyses of the recipient LNT01, donor strains and LNT01 transconjugants from three separate matings are shown in Figures 3–5. LNT01 (recipient), wild type and plasmid-tagged donor strains, and three clones of each transconjugant all exhibited unique restriction banding patterns when digested with *Sma*I, *Xho*I or *Nar*I. PFGE followed by Southern hybridization analyses using the *ermB* probe (intron probe) and the appropriate neurotoxin gene probes showed that the tagged plasmids were transferred to the recipient strains (Figures 3–5).

**Figure 4 pone-0011087-g004:**
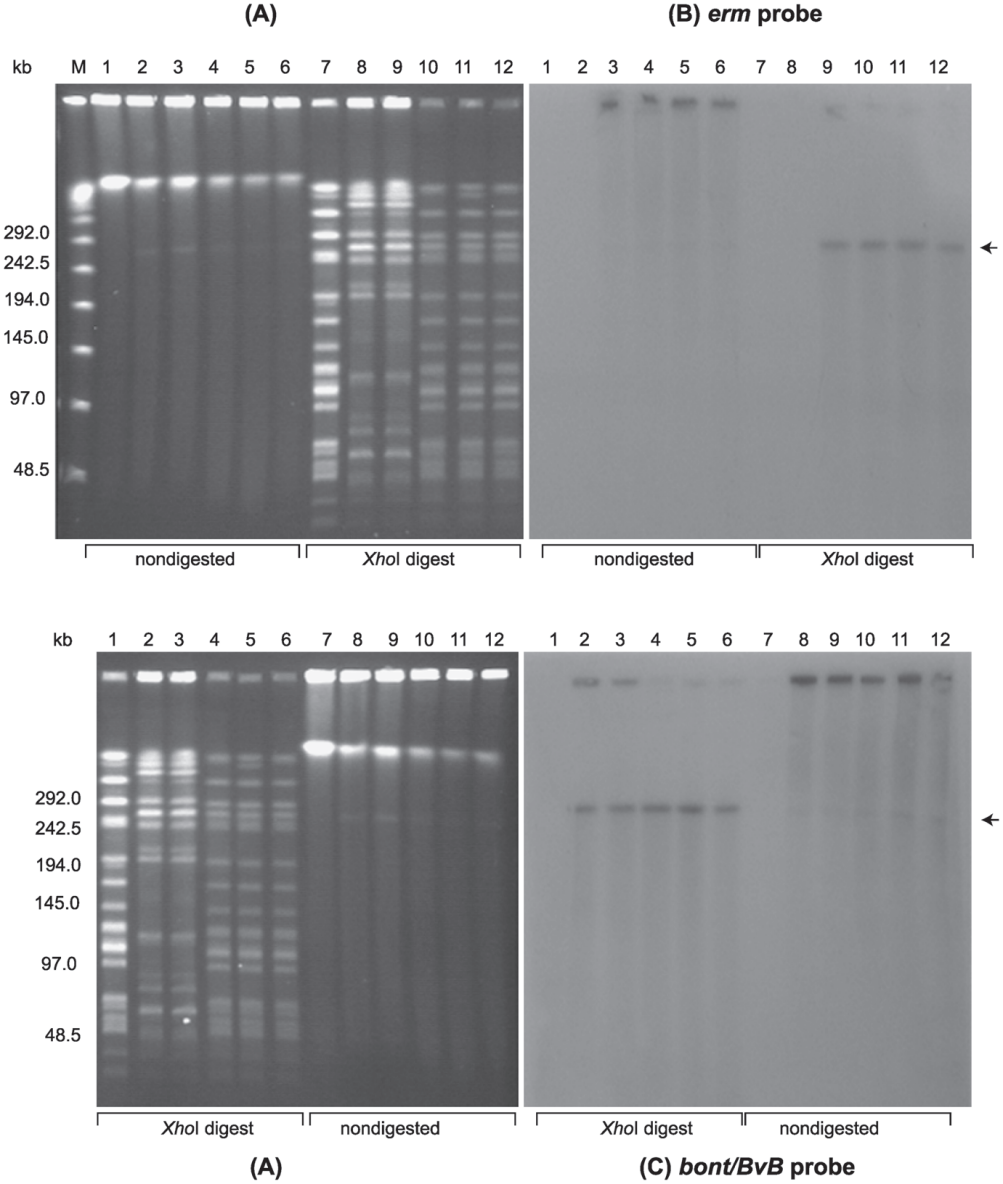
Confirmation of plasmid pCLJ-Erm transfer from *C. botulinum* strain 657BaCT4 to strain LNT01. (**A**) Ethidium bromide stained PFGE of *C. botulinum* strains: LNT01 (Lanes 1 and 7), wild type strain 657Ba (Lanes 2 and 8); 657BaCT4 (Lanes 3 and 9) and LNT01 transconjugants (pCLJ-Erm) (Lanes 4–6 and 10–12); Lanes 1–6, nondigested DNA samples; Lanes 7–12, *Xho*I digested DNAsamples. Lambda PFG Marker (Lane M), New England Biolabs. The position of the pCLJ plasmid is indicated with an arrow. Southern hybridization with: (**B**) the *ermB* probe and (**C**) the *bont/bvB* probe. PFGE conditions: 6V/cm, 12°C, 1–26 s pulse time, 24 h.

**Figure 5:**
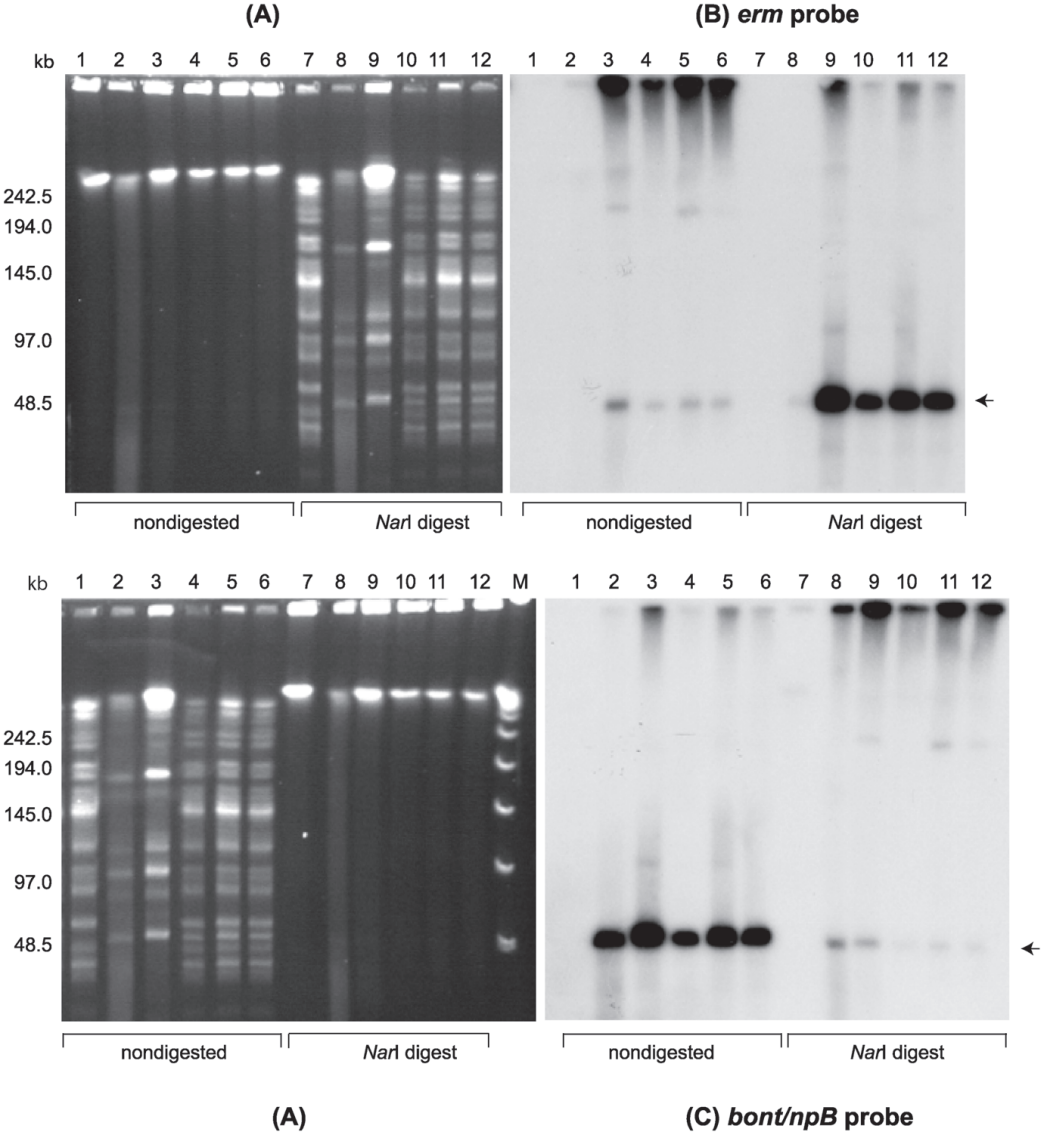
Confirmation of plasmid pCLL-Erm transfer from *C. botulinum* strain Eklund 17BCT11 to strain LNT01. (**A**) Ethidium bromide stained PFGE of *C. botulinum* strains: LNT01 (Lanes 1 and 7), wild type strain Eklund 17B (Lanes 2 and 8); Eklund 17BCT11 (Lanes 3 and 9) and LNT01 transconjugants (pCLL-Erm) (Lanes 4–6 and Lanes10–12). Lanes 1–6, nondigested DNA samples; Lanes 7–12, *Nar*I digested DNA samples. Lambda PFG Marker (Lane M), New England Biolabs. The position of the pCLL plasmid is indicated with an arrow. Southern hybridization with: (**B**) the *ermB* probe, and (**C**) the *bont/npB* probe. PFGE conditions: 6V/cm, 12°C, 1–20 s pulse time, 24 h.

Transfer of pBotCDC-A3-Erm from CDC-A3 (donor) to LNT01 (recipient) is shown in Fig. 3. When PFGE is performed on nondigested *C. botulinum* DNA samples most of the DNA remains trapped in the wells because large circular DNA molecules that are nicked or enzymatically relaxed fail to enter the gel matrix [28]. Linear forms of plasmids are able to migrate through the gel to a position which corresponds to their linear size relative to a reference marker [28]. In addition, a small portion of sheared chromosomal DNA migrating a short distance from the well position is frequently observed in PFGE analysis of nondigested clostridial DNA [17]. The DNA restriction banding pattern of the wild type strain LNT01 (lane 7A) digested with *Sma*I was identical to that of the transconjugants (lanes 10–12A), except that the banding pattern of all transconjugant clones contained an additional band of ∼270 kb. This band corresponds by size to the plasmid, pCDC-A3-Erm in the donor strain (A-lanes 2, 3 and 8, 9). This ∼270 kb band observed in digested (lanes 10–12) and nondigested (lanes 4–6) samples of the transconjugants hybridized with both *bont/A3* (C: lanes 4–6, 10–12) and *ermB* probes (B: lanes 4–6, 10–12). These results confirmed the transfer of the tagged plasmid containing the intron interrupted *bont/A3* gene. The same plasmid band in the donor strains hybridized with the *bont/A3* probe (C: lanes 2, 3, 8, 9), but only the tagged donor hybridized with the *ermB* probe (B: lanes 3 and 9). No hybridization signals were detected with either probe in the recipient strain LNT01. PFGE of digested samples of the transconjugant and donor strains (A: lanes 8–12) showed an increase in the intensity of the ∼270 kb band in the ethidium bromide stained gel as well as produced stronger hybridization signals with the neurotoxin gene (C: lanes 8–12) and *ermB* (B: lanes 9–12) probes, while the hybridization signals at the well positions decreased. This indicated that the plasmid was linearized by the restriction enzyme and migrated into the gel.

**Figure 6:**
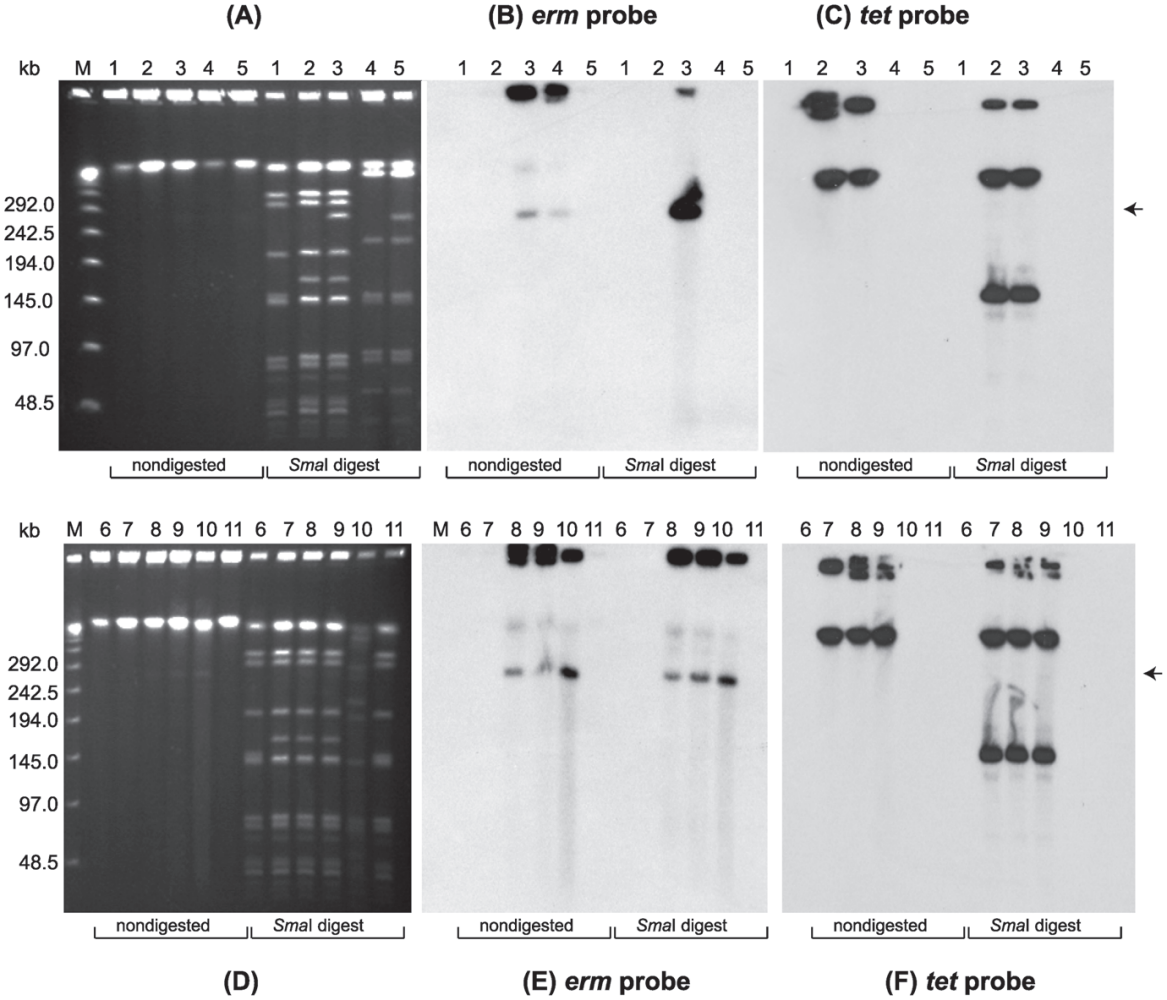
Confirmation of plasmid pCLK-Erm and pCLJ-Erm transfer to *C. botulinum* Hall A-*hyper*-Tn*916* mutant strain. (**A**) Ethidium bromide stained PFGE of *C. botulinum* strains: wild type Hall A-*hyper* (Lane 1), Hall A-*hyper*/Tn*916* mutant (Lane 2); Hall A-*hyper*/Tn*916*/pBotCDCA3-Erm (Lane 3); CDC-A3 plasmid-cured (Lane 4); wild type CDC-A3 (Lane 5). (**D**) Ethidium bromide stained PFGE of *C. botulinum* strains: wild type Hall A-*hyper* (Lane 6), Hall A-*hyper*/Tn*916* mutant (Lane 7); Hall A-*hyper*/Tn*916*/pCLJ-Erm (Lanes 8 and 9); 657Ba-CT4 (Lane 10); wild type Hall A-*hyper* (Lane 11). Nondigested DNA and *Sma*I digests were loaded on the gels as indicated below the lanes. Lambda PFG Marker (Lane M), New England Biolabs. The position of the pBotCDC-A3 and pCLJ plasmids is indicated with arrows. Southern hybridization with: (**B** and **E**) the *ermB* probe; and (**C** and **F**) the *tet* probe. PFGE conditions: 6V/cm, 12°C, 1–26 s pulse time, 24 h.

Similarly, transfer of the ∼270 kb plasmid, pCLJ-Erm, from the donor strain 657BaCT4 to LNT01 (Fig. 4), and the ∼48 kb plasmid, pCLL from Eklund 17BCT11 to LNT01 was confirmed (Fig. 5). Furthermore, transfer of plasmids pBotCDC-A3-Erm, and pCLJ-Erm to Hall A-*hyper*/Tn*916* was also confirmed by PFGE and Southern hybridization analyses (Fig. 6).

### Plasmid Alignments

**Figure 7:**
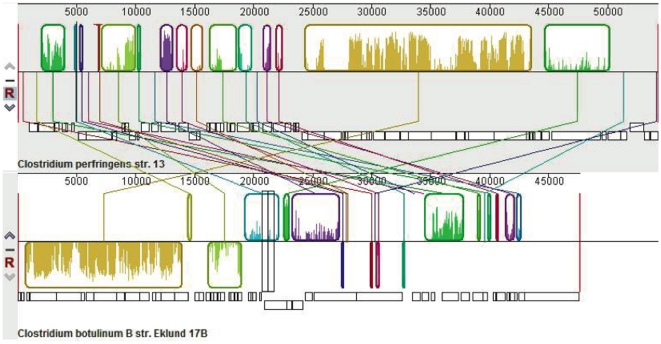
Plasmid alignment of pCLL (*C. botulinum* strain Eklund 17B) and pCP13 (*C. perfringens* strain 13). The alignment has two panels, one for each complete plasmid: pCP13 [top position] and pCLL [bottom position]. The top portions of the panels are composed of colored segments corresponding to the boundaries of locally collinear blocks (LCBs) with lines connecting the homologous blocks in each plasmid. LCBs below a plasmid's centerline are in the reverse complement orientation relative to the reference plasmid (pCP13). The lower portion of the panels represent the predicted open reading frames (ORFs) for the corresponding segments of double stranded DNA with ORFs on top representing top strand and below (bottom strand).

**Figure 8:**
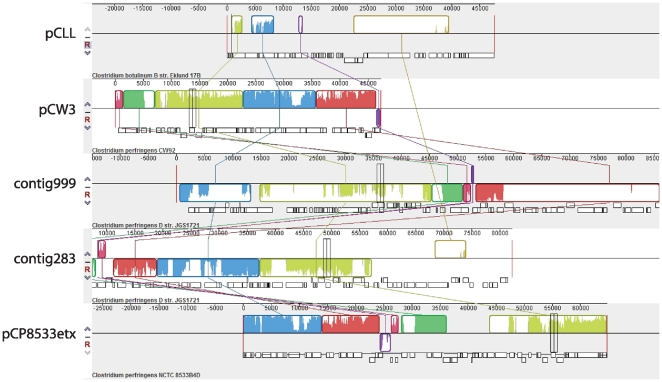
Plasmid alignment of pCLL (*C. botulinum* strain Eklund 17B), pCW3 (*C. perfringens* strain CW92), contigs 1108490430999 and 1108490430283 (*C. perfringens* type D strain JGS1721) and pCP8533etx (*C. perfringens* type B strain NCTC8533B4D). The alignment has five panels, one for each plasmid. The top portions of the panels are composed of colored segments corresponding to the boundaries of locally collinear blocks (LCBs) with lines connecting the homologous blocks in each plasmid. LCBs below a plasmid's center line are in the reverse complement orientation relative to the reference plasmid (pCLL).

The genome alignment tool Mauve [29] was used to generate global alignments of *C. botulinum* plasmid pCLL (strain Eklund 17B) and *C. perfringens* plasmid pCP13 (strain 13). Alignment of plasmids pCLL and pCP13 (Fig. 7) revealed 16 locally collinear blocks (LCBs) with at least some portion of them found in pCP13. The LCB shown in gold (Fig. 7) encompassed a region of 11 ORFs which exhibited the highest degree of sequence homology with similar ORFs found on plasmid pCP13. Mauve was also used to generate global alignments of pCLL and *C. perfringens* plasmids pCW3, pCP8533etx, and two contigs of *C. perfringens* type D strain JGS1721 (gcontig_1108490430283 and gcontig_1108490430999). The alignments revealed several locally collinear blocks (LCBs) with at least some portion of them found in the *C. perfringens* plasmids (Fig. 8). Two neighboring LCBs shown in green and blue were of particular interest because they shared homology with the conjugative *C. perfringens* plasmids. The region that contained the *tcp* locus common to *C. perfringens* conjugative plasmids is represented by the LCB colored blue (Fig. 8). The corresponding blue LCB observed in pCLL shares some sequence homology with this region as indicated in Fig. 8, however this region is truncated. The green LCB of plasmid pCLL contains a gene that encodes for a putative type IV secretion system protein VirD4 (pCLL_0005). Comparison of homologous ORFs of pCLL and *C. perfringens* plasmids is presented in Table 3.

**Table 3:** Comparison of predicted ORFs of pCLL with plasmids of *Clostridium perfringens*.

pCLL locus	Putative function of pCLL gene product	Function of closest *C. perfringens* relative of gene product, strain, and/or identity	Size (aa)	Coding Sequence position
pCLL_0004	Hypothetical protein	Hypothetical protein, *C. perfringens* pCP13, PCP53, and conserved hypothetical, *C. perfringens* E str. JGS1987, CPC_A0335, 53/78 (67%)	78	717–953
pCLL_0005	VirD4 component	TraG/TraD family, *C. perfringens* D str. JGS1721, CJD_A0258, 383/747 (51%)	739	1017–3236
pCLL_0006	Hypothetical protein	Putative membrane protein, *C. perfringens* C str. JGS1495, CPC_A0332, 162/353 (45%), hypothetical protein *C. perfringens* pCP13, PCP50, 162/353 (45%)	711	3241–5376
pCLL_0007	Hypothetical protein	Hypothetical protein, *C. perfringens* pCP13, PCP49, 47/87 (54%)	91	5377–5652
pCLL_0008	Hypothetical protein	Hypothetical protein, *C. perfringens* pCP13, PCP48, 66/124 (53%)	138	5764–6402
pCLL_0009	Hypothetical protein	Conserved hypothetical, *C. perfringens* C str. JGS1495, CPC_A0328, 406/627 (64%)	637	6458–8371
pCLL_0010	Hypothetical protein	Hypothetical protein, *C. perfringens* pCP13, PCP45, 55/161 (34%)	167	8373–9053
pCLL_0011	Probable cell wall-binding protein	Probable cell wall-binding protein, *C. perfringens* E str. JGS1987, AC3_A0050, 224/370 (60%), TcpG, *C. perfringens* C str. JGS1495, CPC_A0146, 83/134 (61%)	389	9114–10283
pCLL_0012	Hypothetical protein	Conserved hypothetical protein, *C. perfringens* D str. JGS1721, CJD_1944, 106/267 (39%)	270	10302–11114
pCLL_0013	Hypothetical protein	Conserved hypothetical protein, *C. perfringens* C str. JGS1495, CPC_A0323, 42/82 (51%)	91	11397–11672
pCLL_0014	Hypothetical protein	Conserved hypothetical protein, *C. perfringens* D str. JGS1721, CJD_A0233, 48/123 (39%)	136	11678–12088
pCLL_0015	Hypothetical protein	Conserved hypothetical protein, *C. perfringens* C str. JGS1495, CPC_A0321, 200/377 (53%)	379	12102–13241
pCLL_0016	Hypothetical protein	Conserved hypothetical protein, *C. perfringens* D str. JGS1721, CJD_A0227, 55/116 (47%)	365	13267–14388
pCLL_0017	Conserved hypothetical protein	Hypothetical protein, *C. perfringens* str. 13 pCP13, PCP34, 31/74 (41%)	171	14453–14968
pCLL_0040	Resolvase/recombinase	Resolvase/Recombinase, *C. perfringens* D str. JGS 1721, CJD_1891, 78/210 (37%)	210	33462–34094
pCLL_0042	Site-specific recombinase resolvase family	DNA-invertase, *C.perfringens* CPE str. F4969, AC5_A0225, 80/191 (41%)	181	34265–34810
pCLL_0045	Replication protein	Replication protein, *C. perfringens* B str. ATCC 3626, AC1_A0161, 164/388 (42%)	446	35980–37320
pCLL_0047	Putative ATPase	Putative ATPase, *C. perfringens* E str. JGS1987, AC3_0198, 145/302 (48%)	297	38545–39438
pCLL_0048	Hypothetical protein	Hypothetical protein, *C. perfringens* E str. JGS1987, AC3_0197, 21/41 (51%)	119	39431–39790
pCLL_0051	Putative LexA repressor	LexA repressor, *C. perfringens B str.* ATCC 3626, AC1_A0290, 34/78 (43%)	235	40570–41277
pCLL_0053	Hypothetical protein	Conserved hypothetical protein, *C. perfringens* B str. ATCC 3626 AC1_A0334, 33/105 (31%)	120	41747–42109
pCLL_0056	Cell wall binding repeat domain protein	Cell wall binding repeat domain protein, *C. perfringens* D str. JGS1721, CJD_0682, 83/183 (45%)	152	42953–47533

## Discussion

Early studies attempting to demonstrate plasmid-associated BoNT genes were unsuccessful, except for discovery of the plasmid-borne BoNT/G gene [15]. The recent finding of plasmids in *C. botulinum* serotypes A and B housing BoNT/A, BoNT/B or both BoNT/A and BoNT/B genes [17], [24] prompted surveys of serotype B strains [18], [19] and dual neurotoxin *C. botulinum* strains producing subtypes Bf, Af and Ab BoNTs [6], [18]. These studies have invigorated interest in the field of plasmid biology in *C. botulinum*. The primary hypothesis of the present study was to determine whether the plasmids encoding BoNT can be transferred to other *C. botulinum* strains.

Here, we have demonstrated the transfer of the native BoNT-encoding plasmids pBotCDC-A3 and pCLJ from two proteolytic strains, and pCLL from a nonproteolytic strain, to other proteolytic *C. botulinum* strains. Our data strongly supports that the mechanism of plasmid transfer is conjugation. Transductants were not obtained when the recipient cells were incubated with filtered donor culture supernatants, supporting that bacteriophages were not involved in BoNT gene transfer. Furthermore, since BoNT gene transfer was not inhibited by the addition of DNaseI, and no transconjugants were obtained during matings in which the donor and recipient cells were separated by a 0.45 µm filter, we conclude that cell-to-cell contact is required for the transfer of these plasmids. Hence, plasmid transfer appears to be due to conjugation or a conjugation-like mechanism rather than by transformation.

Prior to this study, *C. perfringens* was the only *Clostridium* described to harbor plasmids capable of intraspecies conjugative transfer [25], [26], [27], [30], [31]. In this study, conjugative transfer of plasmid pCLL from the nonproteolytic *C. botulinum* serotype B strain Eklund 17B to a proteolytic *C. botulinum* strain, supports interspecies transfer since proteolytic and nonproteolytic groups have long been considered to comprise different *Clostridium* species based on different genomic, genotypic and phenotypic characteristics [2], [9].

Intraspecies conjugal transfer of plasmids in *C. perfringens* has been reported to be a highly efficient process with conjugation frequencies of 10^−1^ to 10^−2^ transconjugants per donor [25], [26], [27]. Conversely, the conjugation frequencies for the *C. botulinum* plasmids tested in this study were much lower ranging from 10^−5^ to 10^−8^ (Table 2). *C. botulinum* strain LNT01 was initially selected as a recipient, since it is nontoxigenic and contained the tetracycline resistance marker for positive selection of transconjugants. Although each of the plasmids was successfully transferred to LNT01, we observed a decrease in the number of donor cells during matings. For example, an approximately 4-log reduction in the number of CFU/ml of donors CDC-A3580s1 and 657BaCT4-2 was observed during matings with LNT01. A possible explanation may be that strain LNT01 produces a bacteriocin (unpublished data) that could inhibit the growth or kill the donor cells. *C. perfringens* strain F4969 was also reported to produce a bacteriocin which interfered with the transfer of plasmid pMRS4969 from this strain to the recipient *C. perfringens* strain because the bacteriocin greatly inhibited or killed the recipient cells [27]. Interestingly, only a 1–2 log reduction of donor (CFU/ml) was observed when the nonproteolytic *C. botulinum* strain Eklund 17BCT11 was used as the donor. To further investigate if a plasmid-endoded bacteriocin affected transfer efficiencies, another *C. botulinum* strain (Hall A-hyper/Tn*916*) that does not contain any plasmids nor a plasmid encoded-bacteriocin similar to those identified in *C. botulinum* strains ATCC 3502 (Acc. No. AM412318) [32] and 213B [33], was tested as a recipient. The plasmid transfer frequency of pCLJ and pBotCDC-A3 into Hall A-*hyper*/Tn*916* increased by at least a log compared to that of LNT01 as a recipient while the transfer frequency of pCLL from the nonproteolytic strain Eklund 17B was similar when both recipients were tested (Table 2). Further studies are required to elucidate the factors and mechanisms affecting conjugal transfer and recipient stability of BoNT-encoding plasmids.

*C. botulinum* strain CDC-A3 is identical to strain Loch Maree based on MLST [34] and PFGE analyses [6], and it is highly likely that the plasmids are identical in both strains. The nucleotide sequences of pCLK (strain Loch Maree, A3), pCLJ (strain 657Ba, A4) and pCLL (strain 17B) genome sequences have been deposited in GenBank [24]. Although plasmids pCLK and pCLJ share significant sequence homology, the sequence of plasmid pCLL is unrelated to pCLJ and pCLK [6], [8]. Detailed sequence analysis of the botulinum gene clusters of plasmids pCLK and pCLJ have been performed, but little emphasis has been given to the functions of other plasmid genes [24]. Most of the ORFs of these plasmids are described as putatively encoding hypothetical or conserved hypothetical proteins. The mechanism for plasmid replication is unknown, because gene homologues involved in typical rolling-circle or theta replication have not been identified on these plasmids [24]. Similarly, the mechanism of plasmid transfer is also unknown, but genes homologous in plasmids pCLK and pCLJ that may be involved in plasmid transfer are suggested by the genome annotations. For example, TraK analogs (CLK_A0294; CLJ_A0213) and TraG/D (CLK_A0293; CLJ_A0212) flanked by hypothetical ORFs, as well as genes that encode for putative type II and type IV secretion system proteins, such as pili, have been described.

Plasmid pCLL of nonproteolytic *C. botulinum* serotype B strain Eklund 17B is ∼48 kb in size and contains 54 putative ORFs. As mentioned above pCLL does not exhibit homology with sequenced plasmids in proteolytic *C. botulinum* strains. Therefore, homology searches of pCLL were performed with genome sequences of other clostridial species. Initially, a nucleotide sequence alignment of pCLL with pCP13 of *C. perfringens* strain 13 was performed using Mauve. Surprisingly, this analysis identified regions of homology between pCLL and pCP13, which are graphically displayed as colored locally collinear blocks (LCBs) (Fig. 7). More detailed BLAST analyses revealed eleven ORFs within the gold-colored LCB (CLL_0004 to CLL_0017) with a range of identity from 34 to 67% with ORFs of plasmid pCP13 (Table 3). However, the function(s) of the identified ORFs in both plasmids is unknown, because these putative gene products are described as hypothetical or conserved hypothetical proteins. Considering that pCP13 is not a conjugative plasmid, further sequence analyses were performed between pCLL and completed sequences of conjugative *C. perfringens* plasmids and draft genome sequences of several *C. perfringens* strains.

Interestingly, two conjugative *C. perfringens* plasmids pCW3 and pCP8533etx as well as two contigs representing potential plasmids in a type D strain were identified by Mauve to contain regions homologous to pCLL (Fig. 8). Mauve revealed two LCBs colored green and blue, which represented regions of pCLL homologous to the *C. perfringens* nucleotide sequences. The blue LCB encompassed the *tcp* (**t**ransfer of **c**lostridium **p**lasmids) locus common to conjugative *C. perfringens* plasmids [31]. Although plasmid pCLL does not seem to contain the entire *tcp* locus it does carry genes that encode for proteins that exhibit 61% (CLL_0011) identity to TcpG (CPC_A0146 of *C. perfringens* C strain JGS1495) [31]. The ORF pCLL_0005 (a putative VirD4 homolog) in the green LCB showed 51% identity to CJD_A0258 (a putative VirD4 component) of *C. perfringens* type D strain JGS 1721. Further BLAST analyses revealed that *C. perfringens* type D strain JGS 1721 contained several ORFs with identities ranging from 37%–51% with ORFs of pCLL (Table 3). *C. perfringens* type D strains carry several plasmids ranging in size from ∼48 to 110 kb. These strains produce both alpha-toxin (*plc* gene) and epsilon-toxin (*etx* gene). The epsilon toxin is ranked third in potency following BoNTs and tetanus neurotoxin [35]. *C. perfringens* type D strains that produce alpha-toxin and epsilon-toxins, but not the enterotoxin (*cpe* gene) or the beta 2 toxin (*cpb2* gene) have been reported to carry the *etx* gene on a plasmid of ∼48 kb [36]. The *etx* plasmid in *C. perfringens* type D strain JGS 1721 also contains the *tcp* locus [36]. The draft genome sequence of *C. perfringens* strain JGS 1721 consists of 221 contigs. The pCLL ORFs shared homology with 16 and 8 ORFs within two of these contigs, gcontig_1108490430999 and gcontig_1108490430283, respectively. Interestingly, gcontig_1108490430283 carries the genes that encode for *etx* and the *tcp* locus.

Overall, homology searches revealed that BoNT-encoding plasmid pCLL of the nonproteolytic *C. botulinum* strain exhibited some degree of homology with both conjugative and nonconjugative plasmids in *C. perfringens*. These results are interesting from an evolutionary perspective, and may warrant further studies on horizontal gene transfer in pathogenic clostridial species.

The presence of BoNT genes on conjugative plasmids in both proteolytic and nonproteolytic strains of *C. botulinum* is highly significant and could facilitate the dissemination of neurotoxin genes to other species of clostridia. It is conceivable that BoNT-encoding plasmids were involved in the transfer of BoNT/E and BoNT/F genes to *C. butyricum* and *C. baratii*. Studies have shown that *C. butyricum* does not possess BoNT-encoding plasmids [37], but considering the suggested mobility of the neurotoxin gene clusters [8], [18], [24], plasmids could be utilized by the organism to mobilize the neurotoxin gene cluster into another bacterial host. Whether or not the plasmid backbones are integrated into the newly acquired host or simply lost is unknown and requires further investigation.

In summary, this study demonstrates for the first time the conjugative transfer of proteolytic and nonproteolytic *C. botulinum* plasmids encoding BoNT genes to other proteolytic *C. botulinum* strains. This work is highly significant in the evolution of pathogenesis of clostridia. Since BoNT is the most potent toxin known, BoNT gene transfer to other bacteria could lead to the generation of new pathogens of high impact, such as emergence of new BoNT-forming clostridia with resistant phenotypes, and strains with higher spore heat resistance than *C. botulinum*. The finding that pCLL of the nonproteolytic *C. botulinum* serotype B strain contains gene regions that are homologous with plasmids in *C. perfringens* is intriguing and illustrates the potential transfer of plasmids to other clostridial species. Further work is needed to elucidate the molecular mechanisms of BoNT-encoding plasmid transfer, their maintenance, and gene expression. The study of the population dynamics of plasmid dissemination to other bacterial hosts in the environment or the human and animal intestine is of concern to human and animal pathogenesis and warrants further study.

## Materials and Methods

### Bacterial Strains

Proteolytic *Clostridium botulinum* strains CDC-A3 (BoNT subtype A3), 657Ba (subtype A4/bivalent B), LNT01 (nontoxigenic, subtype A1), Hall A-*hyper* (subtype A1) and nonproteolytic *C. botulinum* serotype B strain Eklund 17B were obtained from the Johnson laboratory culture collection. *C. botulinum* strain CDC-A3 was originally obtained from the Centers for Disease Control and Prevention (CDC) (Atlanta, GA). MLST and PFGE analyses indicated it was genetically identical to subtype A3 strain Loch Maree [34]. *C. botulinum* strain 657Ba was isolated from a case of infant botulism in 1976 [38]. *C. botulinum* strain Eklund 17B was isolated from marine sediments off the coast of Washington [39]. *C. botulinum* strain LNT01 is a nontoxigenic Tn*916* mutant of the parent strain 62A (subtype A1) [22], [23]. Hall A-*hyper* is a well- characterized subtype A1 strain, which produces high quantities of BoNT/A1 [40]. *Escherichia coli* strains DH10B and CA434 were used for cloning, maintenance and conjugal transfer of the re-targeted ClosTron vectors. All *C. botulinum* strains were maintained as frozen stocks at −80°C in TPGY broth (50 g/liter trypticase peptone, 5 g/liter Bacto peptone, 4 g/liter D-glucose, 20 g/liter yeast extract, 1 g/liter cysteine - HCl, pH 7.4) containing 20% glycerol. Bacterial strains were subsequently cultured anaerobically in TPGY. Mating experiments were conducted on nonselective TYG (30 g/liter Bacto Tryptone, 20 g/liter yeast extract, 1 g/liter sodium thioglycollate) (4% agar) media and then spread plated onto selective TYG (1.5% agar) plates supplemented with the appropriate antibiotics. Antibiotics were used at the following concentrations: cycloserine (250 µg/ml), sulfamethoxazole (76 µg/ml), thiamphenicol (15 µg/ml), tetracycline (10 µg/ml), erythromycin (2.5 µg/ml), chloramphenicol (25 µg/ml in agar plates and 12.5 µg/ml in broth). All bacterial media components and chemicals were purchased from Becton Dickinson Microbiology Systems, Sparks, MD and Sigma-Aldrich, St. Louis, MO.

### Plasmid tagging using ClosTron

The ClosTron mutagenesis system [20], [21] was used to insertionally inactivate *bont/A3*, *bont/bvB* and *bont/npB* of plasmids pBotCDC-A3 (strain CDC-A3), pCLJ (strain 657Ba) and pCLL (strain Eklund 17B), respectively. The computer algorithm available through the Targetron (group II intron) Design Site (http://www.sigma-genosys.com/targetron/) was used to design the PCR primers listed in Table 1 for intron re-targeting of the selected genes *bont/A3*, *bont/bvB* and *bont/npB*. Primers IBS-580|581s, EBS1d-580|581s, IBS-381|382s, EBS1d-381|382s, IBS-420|421s, EBS1d-420|421s and EBS Universal (Table 1) were purchased from Sigma-Aldrich (St. Louis, MO). A two-step PCR reaction was used to generate the 350 bp re-targeted intron. The first step included two separate PCR reactions: one containing the IBS and EBS universal primers and the other containing the EBS2 and EBS1d primers. The intron PCR template supplied in the Targetron Gene Knockout System kit (Sigma-Aldrich, St. Louis, MO) was used as the DNA template. Five microliters of each PCR product obtained in the first PCR reactions were combined and used as the template in a second PCR reaction containing the IBS and EBS1d primers. PCR reactions were performed using the GeneAmp High Fidelity PCR system (Applied Biosystems, Foster City, CA) under the following conditions: initial hold at 94°C for 30s; followed by 20 cycles of 15s of denaturation at 94°C, 30s of primer annealing at 55°C, and 30s of extension at 72°C and then a final 7 min step at 72°C.

The resulting PCR products of 350 bp representing the re-targeted intron were purified by gel extraction (Qiagen) and cloned into the vector pMTL007C-E2 [21] using restriction endonucleases *Hind*III and *BsrG*I by standard cloning techniques [41]. Transformants containing modified ClosTron vectors were selected from *E. coli* strain DH10B based on chloramphenicol resistance, and plasmid DNA was isolated using a plasmid minipreparation kit (Fermentas Inc., Glen Burnie, MD). Plasmids were analyzed by restriction analysis with *Hind*III and *BsrG*I, and the correct sequence of the intron was verified by sequencing using the primer pMTL007-R1 (Table 1). The sequencing primer was purchased from Integrated DNA Technologies, Inc. (Coralville, IA). Sequencing reactions were performed using an ABI PRISM BigDye Cycle Sequencing Ready Reaction kit (Applied Biosystems, Foster City, CA) then purified according to manufacturer's instructions, and analyzed at the University of Wisconsin Biotechnology Center. The nucleotide sequences were aligned and analyzed with sequence analysis software VectorNTI (Invitrogen, Carlsbad, CA). Plasmid DNA from one of the clones containing the correct intron sequence for targeting each BoNT gene was named pMTL007C-E2:Cbo:*bont/A*-580s (*bont/A3*), pMTL007C-E2:Cbo:*bont/bvB*-381s (*bont/bvB*), and pMTL007C-E2:Cbo:*bont/npB*-420s (*bont/npB*) and was transformed into the *E. coli* conjugation donor strain CA434.

Plasmids pMTL007C-E2:Cbo:*bont/A*-580s, pMTL007C-E2:Cbo:*bont/bvB*-381s, and pMTL007C-E2:Cbo:*bont/npB*-420s were transferred to *C. botulinum* strains CDC-A3, 657Ba and Eklund 17B, respectively, by conjugation from *E. coli* donor strain CA434 as previously described [20]. After mating, the bacterial mixture was scraped off of the mating plates, resuspended in 1×PBS (phosphate buffered saline), serially diluted and spread plated onto TYG agar supplemented with cycloserine, sulfamethoxazole (selection of *C. botulinum*) and thiamphenicol (selection for the vectors). Thiamphenicol resistant colonies were purified by re-streaking onto fresh TYG agar supplemented with thiamphenicol. Individual colonies were re-suspended in 1×PBS, serially diluted and plated onto TYG agar containing erythromycin to select for the presence of the spliced Erm-RAM indicating intron integration. Erythromycin resistant colonies were re-streaked onto fresh TYG agar containing erythromycin. Erythromycin resistant clones were replica plated onto TYG containing thiamphenicol to verify plasmid loss by a thiamphenicol sensitive phenotype.

Chromosomal DNA was isolated from randomly selected erythromycin resistant, thiamphenicol sensitive clones as well as from wild type *C. botulinum* strains using the ChargeSwitch gDNA kit (Invitrogen, Carlsbad, CA) following the manufacturer's instructions. Screening of the clones was performed by PCR using the gene specific primers A3KMCT1, A3KMCT2 (*bont/A3*), BVBFCT4, BVBRCT4 (*bont/bvB*), and NPBFCT11, NPBRCT11 (*bont/npB*) (Table 1) designed to anneal to regions flanking the site of intron integration. PCR was performed with AmpliTaq High Fidelity DNA polymerase, buffer and dNTPs (Applied Biosystems, Foster City, CA) using a GeneAmp PCR System 9700 (Applied Biosystems) according to manufacturer's instructions. PCR primers were purchased from Integrated DNA Technologies, Inc. (Coralville, IA). PCR products were visualized on 1% Tris-acetate-EDTA gels, stained with ethidium bromide and photographed using a Gel Imaging System (BioRad, Hercules, CA) with UV transillumination. PCR products were purified using a PCR purification kit (Qiagen, Valencia, CA) according to manufacturer's instructions. The nucleotide sequences of the PCR fragments generated from the wild type and the transconjugant clones were determined using the same primers as for the amplication of the DNA fragments (Table 1). The nucleotide sequences were analyzed as described above.

### Tn*916* Mutagenesis

*C. botulinum* strain Hall A-*hyper* was chosen for Tn*916* mutagenesis to generate tetracycline resistant strains. The genome sequence of Hall A-*hyper* is completed (GenBank Acc: CP000727). This strain does not contain any plasmids. Tn*916* mutant clones of Hall A-*hyper* were generated using the methods for Tn*916* mutagenesis described in Lin and Johnson [22]. This strain containing a tetracycline resistance marker was used as an alternative recipient in the bacterial mating experiments with donors CDC-A3 and 657Ba.

### Mating experiments

Donor and recipient strains were inoculated into TPGY broth from frozen stocks and incubated anaerobically overnight. The strains were subcultured into TPGY containing 2.5 µg/ml erythromycin (donors) and 10 µg/ml tetracycline (recipients). The donors and recipients were passed again in TPGY broth supplemented with antibiotics and incubated anaerobically for 12 h. Each strain was serially diluted to ∼10^4^ to 10^5^ CFU/ml in TPGY broth and incubated until an OD_600nm_ of 0.6 to 0.8. Matings between donors and recipients were performed on solid nonselective 4% TYG agar. Three different donor to recipient ratios (5∶1, 1∶1 and 1∶5) were tested. Aliquots of 1 ml or 200 µl of donor (D) and recipient (R) cells were centrifuged at 3,000×g for 5 min and resuspended in 200 µl of recipient or donor cells, respectively, and spread plated onto 4% TYG agar. The mating plates were incubated right side up for 12 h at 37°C or 30°C depending on the optimal growth temperature of the donor cells. Separate plates spread plated with 200 µl of donor and recipient cells were also included as controls. Sensitivity of plasmid transfer to DNaseI was tested by treating 1 ml of donor cells with DNaseI (100 µg/ml) in a buffer containing 20 mM Tris-HCl, pH 7.5, 1 mM MgCl_2_ and 1 mM MgSO_4_ for 37°C for 60 minutes [42]. On the nonselective agar plate 25 µl of DNaseI (10 mg/ml) and 50 µl of 50 mM MgSO_4_ were added, spread evenly and the plates were incubated at room temperature for 60 minutes. Donor cells incubated for 60 min with DNaseI, were spun down at 3,000×g for 5 min. The supernatant was discarded and the cell pellet was re-suspended in 200 µl of the recipient strain. DNaseI (100 µg/ml) and 1 mM MgSO_4_ were added to the cell mixture and the cells were spread plated onto the TYG agar supplemented with DNaseI and MgSO_4_. The plates were incubated at 37°C for 12 h.

Mating experiments were also performed by separating the donor and recipient cells with a 0.45 µm nitrocellulose membrane to determine if cell-to-cell contact is required for plasmid transfer. In these experiments 200 µl of the donor cell suspensions were spotted in the middle of the plate and spread slightly, and plates incubated until all moisture was absorbed. Then nitrocellulose membrane was placed on top of the donor cells, the plates incubated until the membrane completely adhered to the agar, followed by spreading the recipient cells in the middle of the membrane. The mating plates were incubated right side up for 12 hours at 37°C or 30°C. The recipient cells were then scraped off the surface of the membrane using cell scrapers, the cells were resuspended in the PBS and plated on selective plates.

To assess the possible involvement of bacteriophages in plasmid transfer, donor cell cultures were passed through a 0.45 µm filter (Millipore) and mixed 1∶1 with the recipient cell culture [43]. CaCl_2_ (1mM) was added and the mixture was incubated for 12 h at 37°C.

Following matings the controls and mating mixtures were scraped off of the TYG agar plates and re-suspended in 3 ml of sterile 1×PBS, serially diluted and plated in duplicate on TYG agar supplemented with tetracycline and erythromycin for selection of the transconjugants. Serial dilutions were also spread plated onto TYG supplemented with tetracycline for enumeration of recipients and transconjugants and TYG containing eythromycin for enumeration of donors and transconjugants. The plates were incubated anaerobically for 3 days at 30°C or 37°C. The plasmid transfer frequency was calculated as the number of transconjugants per number of donor cells in matings in which the number of donors was greater than the number of recipients. Transfer frequencies were calculated as the number of transconjugants per number of recipient cells when the recipient counts were greater than the donor cell counts.

Colonies resistant to both tetracycline and erythromycin were re-streaked for isolation onto fresh TYG agar supplemented with tetracycline and erythromycin and kept for further analysis.

### Pulsed-field gel electrophoresis

Confirmation of plasmid transfer was performed by pulsed-field gel electrophoresis (PFGE) of nondigested and digested DNA of the transconjugant, donor and recipient strains. *C. botulinum* strains were inoculated into 10 ml of TPGY and incubated anaerobically at 37°C (proteolytic strains) or 30°C (nonproteolytic strains) to an optical density at 600 nm (OD_600_) of 0.6. One milliliter of formaldehyde (Fisher Scientific, Hampton, NH) was added, and the cultures were placed on ice for 15 to 30 minutes to inhibit nuclease activity. PFGE plugs were prepared as described by Johnson et al. [44].

To increase the visualization of plasmids, pBotCDC-A3-Erm, pCLJ-Erm and pCLL-Erm in the LNT01 transconjugant clones, restriction digests of PFGE plugs were performed using restriction endonucleases chosen to linearize each plasmid. The nucleotide sequences of each plasmid were analyzed using VectorNTI version 10.3 (Invitrogen, Carlsbad, CA) and a rare cutting restriction enzyme that cleaves the plasmid once was selected. Restriction enzymes *Sma*I, *Xho*I and *Nar*I (New England Biolabs), were selected to digest the PFGE plugs of LNT01 transconjugants to assess the presence of plasmids pBotCDC-A3-Erm, pCLJ-Erm and pCLL-Erm, respectively. Restriction digests of the PFGE plugs were performed according to the manufacturer's instructions (New England Biolabs). Two sets of nondigested and digested DNA samples were loaded on the same gel and DNA samples were separated by PFGE in a clamped homogenous electric field system (CHEF-DRII; Bio-Rad, Hercules CA). After the DNA was transferred onto the nylon membrane, the filter was cut in a half, and each portion contained one set of undigested and digested DNA samples. One membrane was hybridized with a neurotoxin gene specific probe, while the other with an erythromycin gene probe.

### Southern Hybridizations

Primers for generation of hybridization probes for *ermB* (ErmF and ErmR); *bont/A3* (A3KMCT1 and A3KMCT2); *bont/bvB* and *bont/npB* (AnyB-F and AnyB-R) are listed in Table 1. These gene probes were generated by PCR amplification with an AmpliTaq High Fidelity DNA polymerase, buffer and dNTPs (Applied Biosystems, Foster City, CA) using a GeneAmp PCR System 9700 (Applied Biosystems) according to the manufacturer's instructions. The PCR products were purified from agarose gels using the Qiagen gel extraction kit (Qiagen, Valencia, CA), and were radioactively labeled with [α-^32^P] ATP using the Megaprime DNA labeling system (GE Healthcare Bio-Sciences, Piscataway, NJ).

The DNA samples separated by PFGE were transferred to a positively charged nylon membrane (Immobilon-NY+, Millipore, Bedford, MA) overnight by downward capillary transfer in 0.4 M NaOH, 1.5 M NaCl. The membranes were neutralized in 2 M Tris-HCl, pH 7.0 for 15 minutes, rinsed with 2×SSC (3M NaCl, 0.3M sodium citrate) and fixed at 80°C for 30 minutes under vacuum.

Hybridizations were performed at 42°C for 16 h in a solution containing 5× Denhardt's Solution, 6×SSPE, 50% formamide, 0.1% SDS, 100 µg/ml herring sperm DNA (Promega, Madison, WI) and ^32^P-labeled probes at ∼2×10^6^ cpm/ml. All hybridization solutions and buffers were prepared according to standard protocols [41]. After hybridizations the membranes were washed twice with 2×SSPE, 0.1% SDS for 5 min each at room temperature and twice with 0.1×SSPE, 0.1% SDS for 30 min each at 42°C. Autoradiography of the membranes was performed for 6–24 h at −70°C using Kodak BioMax MS film with a BioMax intensifying screen (Eastman Kodak, Rochester, NY).

### Plasmid Alignments

Plasmid sequence alignments were performed to determine the relatedness of the plasmid pCLL [Acc. No. CP001057] in *C. botulinum* strain Eklund 17B to plasmids, pCP13 [Acc. No. P003515] of *C. perfringens* strain 13, pCW3 [Acc No. DQ366035] of *C. perfringens* strain CW92, pCP8533etx [Acc. No. AB444205] of *C. perfringens* strain NCTC8533B4D, and two contigs, gcontig_1108490430999 [Acc. No. ABOO01000010.1] and gcontig_1108490430283 [Acc. No ABOO01000017] of *C. perfringens* type D strain JGS1721. Plasmid sequence files with annotations were obtained from NCBI. Plasmid alignments were conducted using progressive alignment option of Mauve 2.3.1 [29] with the default settings. Figures 7 and 8 were generated using the Mauve alignment viewer, which illustrates locally collinear blocks (LCBs) as regions without rearrangements in the homologous backbone sequence. LCBs below a plasmid's center line represent the reverse complement orientation relative to the reference genome (pCP13, Fig. 7; pCLL, Fig. 8). Sequence similarity plots are displayed in the LCBs, and the height of the sequence identity plot reflects the average column entropy for the region of the respective alignment. The NCBI blastp tool was used to compare the amino acid sequences of the pCLL ORFs with ORFs in *C. perfringens* strains.
